# Age and Unplanned Postoperative Visits Predict Outcome after Septoplasty: A National Swedish Register Study

**DOI:** 10.1155/2018/2379536

**Published:** 2018-01-02

**Authors:** Lars Pedersen, Linus Schiöler, Kenneth Holmberg, Cecilia Ahlström Emanuelsson, Johan Hellgren

**Affiliations:** ^1^Department of Otorhinolaryngology, Head & Neck Surgery, Institute of Clinical Sciences, Sahlgrenska Academy, University of Gothenburg, Gothenburg, Sweden; ^2^Department of Occupational and Environmental Medicine, Sahlgrenska Academy, University of Gothenburg, Gothenburg, Sweden; ^3^Department of Otorhinolaryngology, Head & Neck Surgery, Skane University Hospital, Lund University, Lund, Sweden

## Abstract

**Objective:**

To study predictors of symptom relief six months after septoplasty using data from the Swedish National Septoplasty Register.

**Participants:**

This is a retrospective register study of adult patients undergoing septoplasty in Sweden in 2003–2012.

**Outcome:**

Relief of nasal symptoms was analysed in relation to age, gender, size of hospital performing the surgery, addition of turbinoplasty, and unplanned postoperative visits to the hospital due to pain, bleeding, or infection.

**Results:**

In all, 76% of the patients (*n* = 5,865) rated their symptoms as “almost gone” or “gone” six months after septoplasty. With every 10-year increase in the age of the patients, the OR was 1.19, 95% CI 1.15–1.23, for a better result and 1.54, 95% CI 1.38–1.71, if the septoplasty was performed at a county hospital versus a university hospital. If there was no unplanned postoperative visit due to pain, bleeding, or infection, the OR for a better result was 1.6, 95% CI 1.39–1.85.

**Conclusion:**

In this large national cohort of septoplasties, most of the patients felt that their symptoms had gone or almost gone six months after septoplasty. Higher age, surgery at smaller hospitals, and no unplanned visits to the hospital postoperatively predicted a better outcome.

## 1. Introduction

Nasal obstruction is a significant health problem associated with poor sleep and a decreased health-related quality of life [1]. Septoplasty is the recommended surgical procedure to relieve nasal obstruction due to a deviated nasal septum and it is also one of the most common operations to be performed in adults in ear, nose, and throat (ENT) surgery. In some patients, the septoplasty is combined with a resection of the lower turbinate (turbinoplasty) in an attempt to secure increased nasal patency postoperatively. Studies evaluating the surgical result after septoplasty using objective methods such as rhinomanometry or acoustic rhinometry predominantly report a positive effect on nasal air flow and patency [1]. Data on patient-rated relief of symptoms, however, show varying results. Patient-rated relief of symptoms after septoplasty has been estimated at 45–92% in short-term follow-up studies (ranging from* six weeks to six months* after surgery) [2–5]. A few studies have looked at a longer follow-up period after surgery. Siegel et al. found that 71% of patients had improved nasal symptoms* nine months* after surgery [6]. Konstantinidis et al. reported that 45% of patients had improved nasal breathing, 43% reported no change, and 12% reported poorer nasal breathing two to three years after septoplasty [7]. In a Swedish follow-up study 34–70 months after surgery, 53% of the patients reported persistent or worse symptoms [8]. To summarise, both short-term and longer follow-up studies show that a significant number of patients undergoing septoplasty are not relieved of their symptoms.

Studies of the patient-rated outcome in septoplasty show that female gender and previous nasal surgery predict a poorer outcome [6]. Two studies found that the grade of the surgeon was a less important factor [6, 9] and the addition of turbinoplasty to the septoplasty showed a significantly better outcome in one study and no effect in another [10, 11]. Rhinitis, including allergy, nasal congestion, postnasal drip, rhinorrhea, and nasal packing, did not affect the surgical outcome in one study [10] and the inclusion of a preoperative rhinomanometry in selecting patients for septoplasty has shown conflicting results [3, 12]. It appears, however, that patients with high nasal airway resistance preoperatively and a reduction in airway resistance after surgery are more satisfied with the surgical result as would be expected [13].

Although some predictors of a poorer outcome after septoplasty, such as previous septoplasty and the lack of a preoperative rhinomanometry, have been identified, there is still a lack of knowledge relating to why some patients are not relieved of their symptoms after septoplasty. Most of the available data have also been derived from small cohorts and single surgical centres and there is therefore a need for studies analysing patient-rated outcome after septoplasty in large, multicentre populations.

The present study is based on data from the Swedish National Septoplasty Register (SNSR). This is a national quality register for septoplasty that was started in 1997 and around 18,000 septoplasty procedures have been registered there. The main outcome in the register is patient-rated relief of symptoms six months after surgery. In the present study, we have analysed patient-rated relief of symptoms in relation to age, gender, and type of procedure and size of hospital in all available data from the register during a specific time period.

## 2. Material and Methods

### 2.1. Inclusion and Exclusion Criteria

Patients > 18 years of age registered in the Swedish National Septoplasty Register, SNSR, between 2003 and 2012, who had undergone a* primary* septoplasty or primary septoplasty + turbinoplasty with the indication of* nasal obstruction,* were included in the study. All the patients who underwent septoplasty or septoplasty + turbinoplasty within two weeks or less from the decision to perform surgery to the actual septoplasty were excluded to avoid procedures where septoplasty was performed as an emergency procedure following nasal trauma or other acute conditions. All the patients who underwent revision septoplasty were also excluded.

### 2.2. Outcome Variables

Patient-rated relief of symptoms six months postoperatively was analysed in relation to age, gender, type of surgery (septoplasty alone or septoplasty + turbinoplasty), size of hospital or surgical centre where the septoplasty was performed, and whether the patient made any unplanned visits to the hospital within two weeks after the surgery due to nasal pain, infection, or bleeding. Hospitals were divided according to size as* “university,” “county,”* and* “district”* hospitals. Surgical centres within private health care (a minority in Sweden) and smaller surgical centres that are not full hospitals were categorised as* “others.”*

### 2.3. The Questionnaires

The SNSR contains data from three questionnaires filled out in relation to the septoplasty of the patient and the items on the three questionnaires are listed in Table 1. The first questionnaire was filled out by an ENT surgeon who diagnosed the patient and contains data on indication (nasal obstruction) and whether the planned septoplasty was primary or revision surgery. Only patients with the main symptom of nasal obstruction undergoing primary surgery were included in the study and, for instance, patients undergoing septoplasty due to inability to use a CPAP-machine were excluded. The second questionnaire was filled out by the ENT surgeon performing the septoplasty, confirming date of surgery and type of surgical procedure performed (septoplasty alone or in combination with a turbinoplasty). The questionnaire contains no data on specific surgical technique (endonasal, endoscopic, with or without removal of cartilage/bone or nasal packaging), the experience of the surgeon, or the severity of disease. A third questionnaire was mailed to the patient six months after the septoplasty. The patient was asked to rate his/her remaining nasal symptoms six months after surgery according to four categories: “4: my symptoms have gone,” “3: my symptoms have almost gone,” “2: my symptoms remain,” or “1: my symptoms have worsened.” Patients were also asked to report any unplanned visits to the hospital within the first two weeks after the septoplasty due to any of the following complications: nasal pain, bleeding, or infection. The return of a completed questionnaire was regarded as informed consent.

### 2.4. The Register

**Figure 1:**
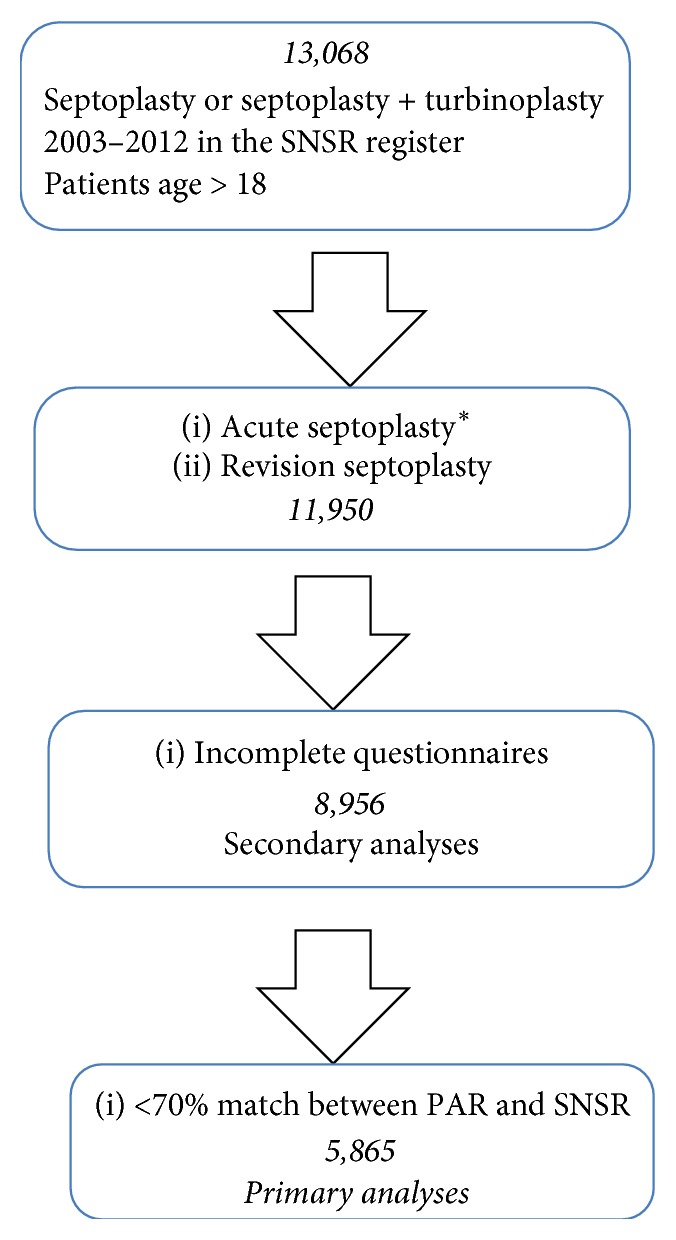
Flow chart of the study population, *N* = 5,865, from the Swedish National Septoplasty Register (SNSR). National Patient Register (PAR). ^*∗*^Septoplasty within 2 weeks of the decision to perform surgery.

The SNSR was started in 1997 and is administered by ENT doctors' professional national organisation: the Swedish Association for Otorhinolaryngology, Head & Neck Surgery (SFOHH, http://www.orlforum.se), in collaboration with the government organisation, the Swedish Association of Local Authorities and Regions (SKL, https://skl.se/tjanster/englishpages.411.html), which is in charge of public health care in Sweden. In this study, we selected a 10-year period from 2003–2012 to collect data from the SNSR, as this period has the best reporting rate in the register. The completeness of the data in the SNSR was validated against the National Patient Register (PAR), where all the surgical procedures performed in Sweden are reported. The PAR includes data on diagnosis, surgical code, and date of surgery and it is administered by the Swedish Board of Health and Welfare (Socialstyrelsen, http://www.socialstyrelsen.se/register/halsodataregister/patientregistret/inenglish). The PAR includes the personal identity number of the patients that enables match analyses to the SNSR. Unlike the SNSR, the PAR does not include any data on surgical outcome. In 2003–2012, each hospital participating in the SNSR, that is, the majority of hospitals in Sweden, had varying levels of completeness of data when compared with the PAR. In the primary analyses of this study, a cut-off of at least 70% completeness (match between the SNSR and PAR) was applied for each hospital and year, *N* = 5,865 (Figure 1). In a secondary analysis, we included all the reported data in the SNSR from 2003 to 2012, *N* = 8,956.

### 2.5. Statistical Analysis

Descriptive statistics are presented as percentages or mean values and range. The age distribution in the different response groups of patient-rated symptom relief is illustrated in a box plot. The association between patient-rated symptom relief and different predictors was analysed using a multivariate ordinal logistic regression model, also referred to as a proportional odds model [14]. The interpretation of the odds ratio (OR) is similar to that of the ordinary logistic model, the difference being that the odds of having the same or a better reported outcome are modelled, rather than the odds of a single event. The study was approved by the Local Ethics Committee in Gothenburg, number 074-15.

## 3. Results

**Figure 2:**
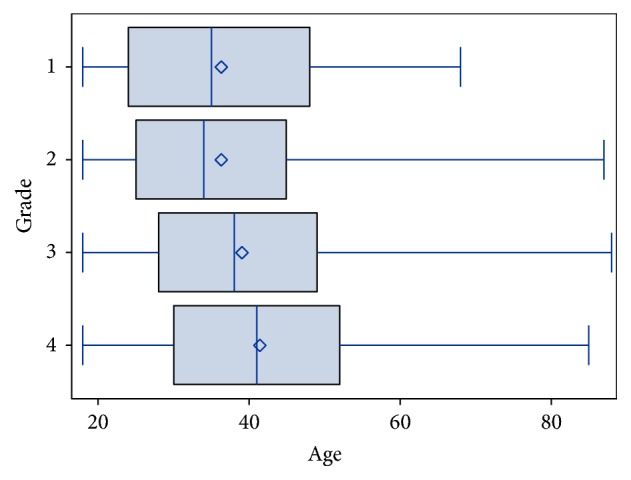
Patient age in relation to patient-rated relief of nasal symptoms six months after surgery. 1 = my symptoms have worsened (*N* = 167), 2 = my symptoms remain (*N* = 1,254), 3 = my symptoms have almost gone (*N* = 2,753), and 4 = my symptoms have gone (*N* = 1,691). Missing data from the third questionnaire (*N* = 2,049).

The patient-rated relief of symptoms six months after surgery and associated factors for the 5,865 subjects in the primary analysis are presented in Table 2. Considerably more men (76%) than women underwent septoplasty during the follow-up period from 2003 to 2012. The majority of the surgical procedures were performed at ENT departments at the largest hospitals, such as county or university hospitals (more than 80%). Patients operated on at the university hospitals were slightly less satisfied than the patients operated on at county or district hospitals. In all, 76% of the patients reported that their symptoms had “almost gone” or “gone.” Almost 3% experienced a worsening of their symptoms. A higher mean age among the patients predicted a better outcome, Figure 2. Unplanned visits due to nasal bleeding, infection, or pain within two weeks of surgery were almost twice as common in subjects reporting “symptoms remain” after six months as for the others. Odds ratios for factors predicting patient-rated outcome are presented in Table 3. An unplanned visit to health care within two weeks of surgery was the strongest predictor of a poorer outcome. A secondary analysis of all the subjects registered in the SNRS between 2003 and 2012 (not taking completeness of data of 70% into account), *N* = 8,956, did not change the result. A total of 2,049 patients did not answer the follow-up questionnaire after six months. The data for the “nonresponders” are shown in Table 4.

## 4. Discussion

To our knowledge, this is the first study to analyse the surgical outcome and predictors of septoplasty in a large, multicentre, national sample of patients undergoing septoplasty and, in all, 76% were satisfied with the result. The results also show that if the patient is older and the surgery is performed outside a university hospital, the outcome is better. Having to make unplanned visits to health care due to nasal bleeding, infection, or pain within two weeks of surgery was the strongest factor associated with a poorer outcome.

One advantage of this study is the large number of patients included from several surgical centres in one country and over a 10-year period, resulting in a total of almost 6,000 patients. The national base of the study has also enabled us to make a comparison of the surgical outcome between hospitals of different sizes in a uniform public health care system. One important finding was that a higher patient age predicted a significantly better outcome six months after surgery. For each 10-year increase in age, there was an increase in the odds of 1.2 of having a one level better subjective score (e.g., symptoms gone versus symptoms almost gone). To our knowledge, this has not been reported before and it may be a factor to take into account when planning septoplasty and informing patients about the expected outcome of the surgery. The reason for this effect of age is not clear and it is possible to speculate that younger patients may have different expectations than older patients, based on different previous life experiences. Healing conditions after surgery can differ with age, as changes in the cartilage have been described with increasing age [15]. Higher age has also been associated with larger nasal cavities measured with acoustic rhinometry, which could affect the potential for mechanical improvement in a positive way with increasing age after surgery [16]. A study of 7,000 post office employees in Japan showed that younger people report more nasal obstruction than older persons, which may reflect more concomitant disease, such as upper airway inflammation [17]. Older patients may also have alternate indications for a septoplasty such as inability to use nasal CPAP, but those patients were excluded in this study.

In our study, we were unable to adjust postoperative outcome to allergic rhinitis, asthma, or chronic rhinosinusitis (CRS), although one previous study has indicated that this did not affect the outcome after septoplasty [10].

In agreement with previous studies, we found that almost 3% of the patients reported worse symptoms after surgery. Interestingly, there was no difference between gender or between septoplasty with or without turbinoplasty, while surgery at a university hospital and reporting unplanned visits due to nasal bleeding, infection, or pain increased the risk of a poor result. All types of surgery have an inherent risk of failure due to the risk of complications. Unplanned visits to health care due to nasal bleeding, infection, or pain indicate complications which could result in defective healing. The finding that surgery at a university hospital was associated with a poorer outcome than surgery at county or district hospitals is interesting. We are not aware of any previous studies from large cohorts reporting similar results. It is well known that more difficult cases tend to accumulate at university clinics through referrals from other centres and this could explain the higher risk of a poorer outcome. University clinics are also located in larger cities and surgery there may come with higher expectations on the part of patients.

This study confirmed that men are overrepresented in septoplasty, which has been shown in previous studies. The reason for this is unclear. It could be because men have previously been more involved in sports and activities associated with a risk of acquiring a deviated septum [9, 18].

This study includes data from 37 surgical centres in Sweden and health care in Sweden is predominantly public. It is interesting to see that the variation in results from the different centres is comparatively small, despite different geographical locations and community sizes. A large variation in the patient-rated outcome from the different centres would have indicated that a specific surgical technique or the surgical skill at one centre would have been of major significance to the patient-rated outcome, but that was not the case. It should be noted, however, that these data are lacking in the register and thus not included in the analyses. Furthermore detailed studies are needed to evaluate their significance for the surgical result. The mean positive result of 76% completely or partially resolved symptoms is in good agreement with the previous international data from smaller cohorts and restricted geographical areas. The reason why a quarter of the patients undergoing septoplasty are unhappy with the surgical result remains unclear. It could indicate that the patient selection and the expectations of the result are unrealistic, as studies of nasal air flow and resistance after septoplasty predominantly report a good outcome and are not completely in agreement with the subjective result.

The addition of a turbinoplasty is a measure designed to increase nasal patency and it has previously been shown to be beneficial in combination with septoplasty, but, in our large study cohort, we were unable to confirm this. The flow mechanics of the nasal cavity are still not fully understood and, even though the removal of tissue from the nasal passage per se increases the nasal cross-sectional area and volume, it may not necessarily increase nasal air flow. It may also be that the addition of a turbinoplasty is made predominantly in patients with a worse nasal obstruction, which could be a complicating factor affecting the final outcome.

The present study has several weaknesses. Register studies are sensitive to response rate, which can introduce selection bias. The third questionnaire was answered by 74% of all the patients in the register. The responders were significantly younger, had a higher representation of males, and underwent surgery at university hospitals to a greater extent than the nonresponders.

In a strictly questionnaire-based evaluation of septoplasty, there is a risk of missing important effects on objective air flow and resistance that were not assessed here. Another limitation of this retrospective register study is that we have not been able to stratify the different surgeons, surgical techniques, or severity of disease, as these data were not included in the original questionnaire.

The patient evaluation of symptoms was made six months after the septoplasty, which is a follow-up period that has been used in several previous studies of the subjective outcome after septoplasty and thus enables comparison with these data. Symptoms of nasal obstruction may, however, change over time. One study found that a significant number of patients with high nasal airway resistance after decongestion on at least one side, evaluated for septoplasty, had reduced nasal symptoms and nasal airway resistance when reassessed after eight years using a wait-and-see policy [19]. Relieving upper airway obstruction by septoplasty is still an important procedure to improve health-related quality of life and sleep for many patients. The accurate identification of cofactors that could potentially increase patient-rated satisfaction after septoplasty should therefore be further examined, as a quarter of the patients are not sufficiently relieved of their symptoms.

## 5. Conclusion

To our knowledge, this is the first study to analyse surgical outcome and predictors of septoplasty in a large, multicentre national sample. The results show that the majority of patients experienced a relief of nasal symptoms six months after surgery. Higher age and surgery at smaller hospitals predicted a better outcome. Careful patient assessment of and information about risk factors associated with surgery are important in septoplasty.

## Figures and Tables

**Table 1:** Items in the three questionnaires registered in the Swedish National Septoplasty Register between 2003 and 2012.

	Questionnaire 1	Questionnaire 2	Questionnaire 3
Reporting	ENT surgeon taking decision on septoplasty	ENT surgeon performing septoplasty	Patient

Time of report	When surgery was decided on	At surgery	Six months after surgery

Parameters	Date of decision – septoplasty	Date of septoplasty	Remaining nasal symptoms
	Gender of patient	Date of discharge	
	Age of patient		
	Main indication: nasal obstruction or other		
	Planned type of operation: primary septoplasty or re-operation septoplasty with or without turbinoplasty	Type of operation performed: septoplasty with or without turbinoplasty	Unplanned visits due to nasal pain, infection or bleeding

**Table 2:** Patient-rated relief of nasal symptoms six months after septoplasty or septoplasty + turbinoplasty. % (*n*). *p* values for trend. S = septoplasty, size of hospital where surgery was performed (university, county, district, other), unplanned visits within two weeks of surgery due to nasal pain, infection, or bleeding.

Nasal symptoms	(1) worsened % (*n*)	(2) remain % (*n*)	(3) almost gone % (*n*)	(4) gone % (*n*)	3 + 4 %	Total % (*n*)	*p*
Mean age, years (SD)	36,3 (13.7)	36,3 (13.2)	39,0 (13.6)	41,4 (14.1)		39,1 (13.8)	<0.0001

All	3 (167)	21 (1254)	47 (2753)	29 (1691)	76	100 (5865)	

Male	3 (127)	22 (987)	47 (2084)	28 (1250)	75	76 (4448)	0.007
Female	3 (40)	19 (267)	47 (669)	31 (441)	78	24 (1417)	

Septoplasty	3 (124)	22 (926)	46 (1958)	29 (1225)	75	72 (4233)	0.4
S + turbinoplasty	3 (43)	20 (328)	49 (795)	28 (466)	77	28 (1632)	

University hospital	3 (83)	24 (578)	50 (1223)	23 (550)	73	41 (2434)	<0.001
County hospital	2 (60)	19 (445)	44 (1030)	35 (815)	79	40 (2350)	
District hospital	2 (18)	22 (189)	44 (381)	32 (273)	76	15 (861)	
Other hospital	3 (6)	19 (42)	54 (119)	24 (53)	78	4 (220)	

Unplanned visit	5 (42)	27 (204)	46 (341)	22 (162)	68	13 (749)	<0.001
No unplanned visit	3 (125)	20 (1050)	47 (2412)	30 (1529)	77	87 (5116)	

**Table 3:** Multivariate logistic regression of predictors in relation to outcome after septoplasty presented as odds ratios including age (+10 years), unplanned visit within two weeks of surgery (yes or no), surgical technique (septoplasty versus septoplasty + turbinoplasty), gender, and size of hospital (university versus county, district, and other) with 95% confidence intervals.

Odds ratio estimates and Wald confidence intervals			
Label	Estimate	95% confidence limits	
Age units = 10	1.19	1.15	1.23
Unplanned visit no versus yes	1.61	1.39	1.85
Septoplasty without turbinoplasty vs with turbinoplasty	0.97	0.87	1.08
Gender F versus M	1.16	1.03	1.29
Hospital, district versus university	1.37	1.18	1.58
Hospital, county versus university	1.54	1.38	1.71
Hospital, other versus university	1.19	0.92	1.54

**Table 4:** Comparison between responders and non-responders in relation to age, gender, type of surgery (septoplasty with or without turbinoplasty), and size of hospital.

	Nonresponders % (*n*)	Responders % (*n*)	*p*
			
*Age*, mean years (max-min)	34 (18–78)	39 (18–88)	<0.0001
*Gender*, female versus male	20 (418/1631)	24 (1417/4448)	<0.0005
*Septoplasty with versus without turbinoplasty *	33 (669/1380)	28 (1632/4233)	<0.0001
*Hospital size* other/district/county/university	6.11.38.44 (127/232/785/905)	4.15.40.41 (220/861/2350/2434)	0.0002
